# Tellurium Corrosion of Type 304/304L Stainless Steel, Iron, Chromium, and Nickel in High-Temperature Liquid Sodium

**DOI:** 10.3390/ma16206798

**Published:** 2023-10-21

**Authors:** Yi Xie

**Affiliations:** School of Nuclear Engineering, Purdue University, West Lafayette, IN 47907, USA; xie90@purdue.edu

**Keywords:** liquid sodium, tellurium attack, stainless steel, sodium-cooled nuclear reactor

## Abstract

Investigating tellurium (Te) corrosion on structural materials is crucial for sodium-cooled fast reactors (SFRs) due to radionuclide presence and knowledge gaps. In this study, Type 304/304L stainless steel (SS304), chromium (Cr), iron (Fe), and nickel (Ni) samples were immersed in low-oxygen environments with Te in liquid sodium at 773 K for 30 days. At 10 ppm oxygen, SS304 showed multiple oxide layers, including a compact NaCrO_2_ interlayer and porous Na-Fe-Ni-O outer layers. Tellurium penetrated through the porous layers but was hindered by the NaCrO_2_ interlayer. At 0.01 ppm oxygen, Cr had no oxide layer, while Fe and Ni had unstable ones. Tellurium-induced pitting was deeper in Fe and Ni compared to Cr. Oxygen levels and Cr composition are critical factors affecting stable oxide compound layer formation and mitigating Te-induced pitting.

## 1. Introduction

Radionuclide release in the primary sodium coolant due to fuel and cladding failures is one of the many issues associated with the development and deployment of sodium-cooled fast reactors (SFRs) [1]. In a fuel damage accident, most fission fragments might release from the failed fuel into the primary sodium loop and remain within or be absorbed by the sodium-wetted metal surfaces, while the others flow with the helium cover gas over the liquid sodium in the reactor pool to gaseous storage tanks. In the primary sodium coolant, due to the high retention of fission products (FPs), the chemical interactions of radionuclides with structural materials, including cladding, primary coolant pump, intermediate heat exchanger, and the reactor vessel are of critical concern. Among the radionuclides, the FPs, including cesium (Cs), rubidium (Rb), iodine (I), tellurium (Te), antimony (Sb), barium (Ba), and strontium (Sr), have been detected in the primary sodium under both normal and abnormal operation conditions [2,3]. These FPs are all soluble in liquid sodium, although their solubilities at elevated temperatures are not yet fully understood. There exists a significant gap in our understanding of the consequences associated with the release of radionuclides into the primary sodium coolant.

Tellurium emerges as a key radionuclide released during fuel fragmentation, and its presence has been routinely observed in the primary sodium coolant under various operational conditions [2,3]. Tellurium can induce embrittlement through diffusion and reactions with steel materials [3,4]. Although Te has a good retention capability in the fuel [5], a notable quantity of Te has been discovered in the primary sodium coolant through adsorption or plateout on the primary system structures. This phenomenon is attributed to the high solubility of Te in liquid sodium. It is worth noting that tellurium resides in the same periodic table column as oxygen (O), and as a result, it exhibits similar corrosion property and diffusion behavior as oxygen. Zhang et al. [6] found that the among a number of alloy elements, such as Fe, Cr, Ni, Mo, Nb, Mn, Co, and others, Te exhibits relatively higher diffusivity. This underscores tellurium’s tendency to preferentially diffuse and react with alloy elements, mirroring the behavior of oxygen.

Prior to the recognition of Te corrosion behavior, the preliminary attentions were focused on the molten salt reactor environment, where Te is a common fission product dissolved within the molten salt or fuel salt. Rosenthal et al. [7] found that Te is one of the most corrosive fission products in the molten fuel salt environment, with the capability to disrupt protective oxide layers on structural components. In the fuel salt environments, Te can diffuse into the grain boundaries of structural components containing Ni and Fe, leading to embrittlement [7]. Furthermore, tellurium’s reactive and penetrative behavior has been observed in various conditions. Jia et al. [8] observed the formation of reaction products containing Ni_3_Te_2_, when solid Te was in contact with pure Ni, with the depth of Te penetration increasing with rising temperatures from 773 K to 1273 K. At temperatures between 773 K and 1173 K, Te diffusion into Ni predominantly occurred along grain boundaries. Similarly, in Ni-based alloys with trace alloying elements, Wu et al. [9] noted the formation of telluride scales on the alloy surface when in contact with solid Te, accompanied by Te diffusion along grain boundaries and the formation of surface cracks. In these cases, Te enrichment was observed with M_6_C carbides rather than at grain boundaries. The interaction between telluride scales and metal carbides significantly contributed to enhanced Te corrosion and cracking in the tested Ni-based alloy.

Beyond the preliminary investigations, as mentioned above, our current understanding of Te corrosion in liquid sodium on the main structural materials, including stainless steels, remains limited. In contrast to the molten or fuel salt environment, liquid sodium exhibits a distinct characteristic: alloy elements, primarily Fe, Cr, and Ni, react with Na to form Na-M-O compounds on the surface of solid metal, contingent upon the oxygen potential. The stoichiometry and stability of Na-M-O phases are dictated by the oxygen potential and temperature within the environment. Barker et al. [10] found that the stable compound Na_4_FeO_3_ was exclusively formed on Fe and stainless steel samples only at the higher oxygen levels (>0.4 wt.% or 4000 ppm, 873 K). In contrast, Cr formed the stable compound NaCrO_2_ even at extremely low oxygen levels (20–6200 ppm, 873 K). The formation of a stable Na-Ni-O compound was not observed due to the considerably lower oxygen concentration levels tested compared to the oxygen potential threshold. Bhat and Borgstedt [11] also indicated that the order of oxygen potentials for forming stable Fe-, Cr- and Ni-sodium oxide compounds is Ni >> Fe > Cr. The oxygen potential at the liquid sodium–solid metal interface significantly influences the formation of Na-M-O compounds, determined by the oxygen concentration and the standard free energy of formation of the respective metal oxide. When the oxygen potential in liquid sodium is lower than that in the solid metal, oxygen dissolves into the liquid sodium; conversely, when the oxygen potential is higher, oxygen reacts with the solid metal. Thus, Cr is capable of forming a stable oxide compound (NaCrO_2_) at low oxygen levels, while the Fe oxide compound (Na_4_FeO_3_) requires higher oxygen concentration levels. The formation of a stable Ni oxide compound (Na_2_NiO_2_) is contingent upon oxygen concentration levels significantly higher than those required for chromium’s and iron’s stable oxide compounds.

In light of the above, the presence of Te in liquid sodium introduces complexities into oxidation and corrosion processes, particularly due to tellurium’s ability to penetrate oxide layers and diffuse into metal substrates. Nevertheless, to the best of our knowledge, this process in a specific prototypic environment has not been documented in the available literature.

The present work studies the Te corrosion in liquid sodium, focusing exclusively on corrosion effects. The study excludes irradiation effects and employs stable isotopes. Type 304/304L stainless steel (SS304), a common structural material of sodium reactor core vessels and coolant piping, was chosen for study. Iron, nickel, and chromium, the main alloying elements, were also investigated. These metals were immersed in the liquid sodium containing 1 mol% Te at 773 K. Post-test microstructure characterization was conducted through scanning electron microscopy (SEM), with a particular emphasis on cross-sectional analysis. The findings revealed tellurium’s penetration into the metal substrate, exhibiting distinct behaviors in SS304, Fe, Ni, and Cr.

## 2. Materials and Methods

### 2.1. Materials

The test samples included 2.6 mm diameter rods made of SS304 with ASTM A580 specifications, 5 mm diameter rods composed of high-purity Fe (99.995%) and Ni (99.999%), and a 5 mm cube of Cr (99.95%). These materials were obtained from Alfa Aesar. For the corrosion testing, solid sodium bulk of varying purities was employed. One type, used for the SS304 samples, characterized by a purity level of 99.95%, arrived sealed in a quartz tube from Alfa Aesar. The other type, used for the Fe, Ni, and Cr samples, had a purity of 95% and was sealed within a plastic container upon receipt. The Te utilized in these experiments was in the form of lumps with a purity of 99.999%. The mole fraction of Te to Na was 1 to 100, approximating the solubility limit of Te in liquid sodium at a temperature of 773 K [12,13]. To prevent oxidation, all chemicals were meticulously weighed using an analytical balance within an argon-filled glovebox.

### 2.2. Experimental

The test conditions of each sample are detailed in Table 1. Sample preparation and corrosion testing were conducted within argon-filled gloveboxes to prevent oxidation. Oxygen levels in the gloveboxes were monitored using the oxygen sensor in the glovebox system. The test oxygen concentrations, specifically 10 and 0.01 ppm, were deliberately maintained below the oxygen solubility threshold in the liquid sodium (which is 22.08 ppm at 773 K [14]). To ensure effective sealing and prevent sodium vapor leakage, a custom-designed titanium crucible with a threaded titanium lid and a high-temperature-resistant gasket was employed. The inner volume of the crucible was 10 mL, providing ample space to accommodate approximately 3 g of liquid sodium. This experimental setup successfully maintained liquid sodium at a continuous temperature of 773 K for a duration of 30 days, with no observable vapor leakage, as corroborated by the measurements of crucible weight before and after heating.

In order to maintain consistent surface roughness among pristine samples, the samples were sectioned to a length of 5 mm and then ground using 600-grit SiC sandpaper. Subsequently, each sample was placed in the crucible along with a mixture of 3 g of sodium (Na) and tellurium (Te). The liquid sodium completely immersed the metal sample during the heating process. Following the heating cycle, the crucible was removed from the furnace and allowed to cool within the glovebox. The metal samples were then cleaned using 2-propanol (isopropyl alcohol) to dissolve any sodium residues on their surfaces.

The surface and cross-section of post-test samples were analyzed using SEM. For cross-section analysis, the samples were securely mounted using epoxy resin. The samples were abrasively ground with SiC sandpapers of 600, 800, and 1200 grit, followed by polishing with polycrystalline diamond suspensions of 1 µm. The mounted samples were coated with carbon using a sputter coater prior to the SEM analysis.

### 2.3. Microstructure Characterization

Analysis was conducted using a SEM equipped with a silicon drift energy dispersive X-ray spectrometer (EDS). The SEM was operated at an accelerating voltage of 20 keV. Spectra were collected over an energy range of 0–20 keV, which covers characteristic X-ray energies from all analytes. Spectra were quantified using so-called “standardless” analysis, which uses a stored library of reference spectra to quantify unknown spectra rather than physical standards. This method is generally accurate to a few at./at. % range, depending on sample and microscope observation conditions.

## 3. Results and Discussion

### 3.1. SS304

**Figure 1:**
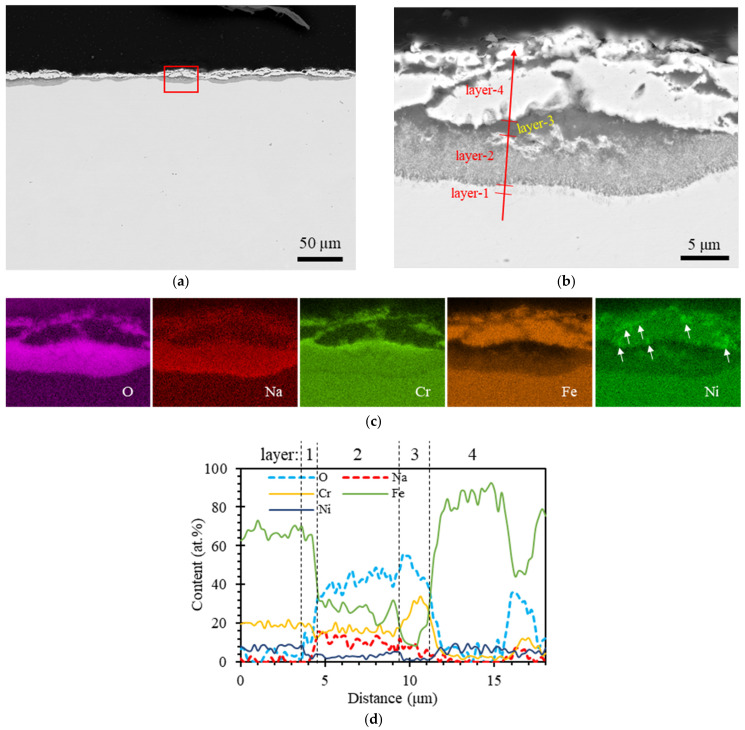
Overview cross-section SEM backscattered electron (BSE) image of post-test SS304 sample (**a**) with a red rectangle indicating the magnified image shown in (**b**). (**c**) shows EDS mapping of elements O, Na, Cr, Fe, and Ni corresponding to the area in (**b**). White arrows represent Ni-rich precipitates. (**d**) presents the EDS line spectrum corresponding to the location in (**b**).

The cross-section of the post-test SS304 sample, with EDS line spectrum, is shown in Figure 1. The oxide layers are roughly 10–20 µm, and no tellurium was detected within these oxide layers. From the SS side to the Na side, four distinct oxide layers were observed, as shown in Figure 1b. Layer-1 was relatively thin (~1 μm), characterized by an increase in Na and O content, formed as a result of inward diffusion. Conversely, there was a reduction in the levels of Fe and Ni due to outward diffusion. Layer-2 was ~5 µm thick, with higher concentrations of Na and O but substantially lower Fe and relatively reduced Ni and Cr compared to layer-1. This was due to its proximity to the Na side, where Na and O diffusion occurred. Iron, Ni, and Cr levels were reduced as they diffused towards the Na side. Layer-3 had a notably higher Cr content compared to the other layers, while Fe and Ni concentrations were relatively lower. This indicates Cr diffusion from layer-2 and its deposition in layer-3. Meanwhile, Fe and Ni continued to diffuse towards the Na side. Layer-4 was distinguished by significantly higher Fe and Ni levels and much lower Cr content compared to the other layers. Additionally, Ni-rich precipitates were present in this layer, as highlighted by the white arrows in Figure 1c. This suggests that Fe and Ni diffused from layers 1 to 3 and were deposited in layer-4. Only a small amount of Cr diffused into layer-4, as the majority of Cr was deposited in layer-3.

**Figure 2:**
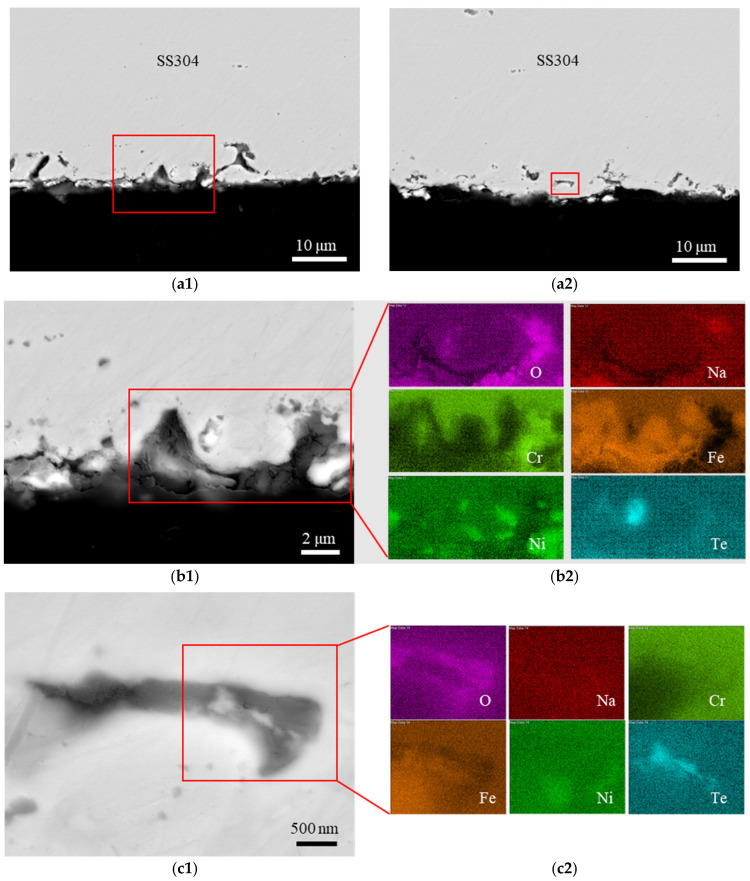
Overview cross-section SEM BSE images of SS304 sample (**a1**,**a2**) each with a red rectangle indicating the magnified images shown in (**b1**,**c1**). The elemental mapping of O, Na, Cr, Fe, Ni, and Te is shown in (**b2**,**c2**), corresponding to the rectangles shown in (**b1**,**c1**), respectively.

In addition to the oxide layers, other locations on the sample were attacked by Te, as shown in Figure 2. These sample surface areas were not covered by oxide layers; instead, they exhibit Te-induced pits about 10 µm deep. Most of these pits were connected to the surface, although some were not, such as the pits shown in Figure 2(c1). Importantly, tellurium was detected within the voids of these pit defects, as shown in Figure 2(b2,c2). In areas where Te concentration was notable, sodium, oxygen, and alloying elements (Fe, Cr, and Ni) were not enriched. The elemental mapping of Cr and O revealed their enrichment at the same locations, consistent with layer-3 in Figure 1. Similarly, the elemental mapping of Fe and O showed that the Fe-enriched areas were depleted of O, aligning with layer-4 in Figure 1. Furthermore, Ni-rich precipitates were dispersed on the edge of the sample, being identical to the Ni-rich precipitates in layer-4 in Figure 1. These distributions indicated that the area was initially oxidized prior to the Te-induced pitting. Therefore, both pieces of evidence indicate that Te is the primary element responsible for disrupting the oxide layers, penetrating the steel substrate, and inducing corrosion.

### 3.2. Chromium

**Figure 3:**
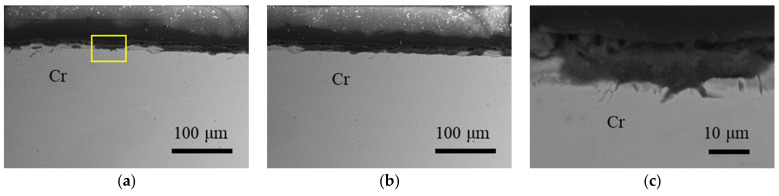
Overview cross-section SEM BSE images of post-test Cr sample in (**a**,**b**), with a yellow rectangle indicating the magnified image shown in (**c**).

The cross-section of post-test Cr sample is shown in Figure 3. There is a significant absence of oxide layers in this sample due to the low oxygen concentration test condition. Compared to the SS304, Fe, and Ni samples, the Cr sample exhibited fewer instances of pitting, and the observed pits tended to be shallower in depth. Remarkably, even the relatively wider and deeper pit displayed in Figure 3c, despite its depth of approximately 5 µm, stands as one of the most notable instances of pitting on the sample. This conspicuous lack of pitting in the Cr sample strongly suggests that Te does not penetrate the surface to the same extent as observed in the SS304, Ni, and Fe samples.

### 3.3. Iron

**Figure 4:**
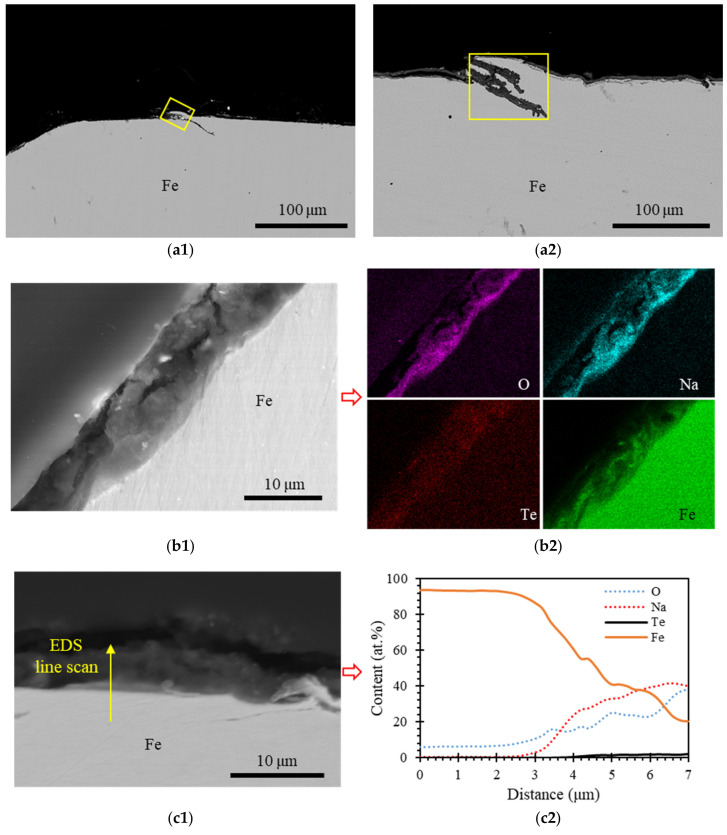

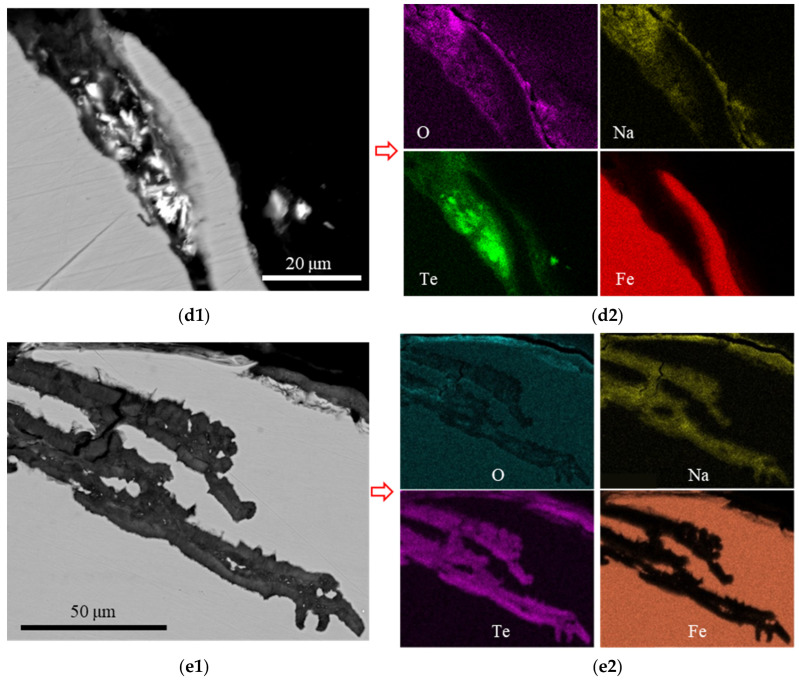
Overview cross-section SEM BSE images of the post-test Fe sample, as depicted in (**a1**,**a2**), each featuring a red rectangle highlighting the magnified image displayed in (**d1**,**e1**). EDS mapping of elements O, Na, Te, and Fe is presented in (**d2**,**e2**), corresponding to the regions of interest delineated in (**d1**,**e1**). (**b1**,**c1**) provide views of the oxide layers along with EDS mapping shown in (**b2**), accompanied by line spectrum data in (**c2**).

The cross-section of the post-test Fe sample with EDS mapping and a line spectrum is shown in Figure 4. The oxide layer thickness is around 10 µm, which is thinner compared to the oxide layer observed in SS304. This oxide layer is composed of O, Na, and Fe, with a low content of Te. When transitioning from the Fe side to the liquid sodium side, the oxide layer exhibited an increase in O and Na content, while experiencing a decrease in Fe content. These compositional changes suggest inward diffusion of O and Na and outward diffusion of Fe. As shown in Figure 4(c2), the content of Te within the oxide layer was less than 2 at. %, falling below the detectable limit of EDS data. Therefore, the oxide layer contained a negligible amount of Te. These characteristics of oxide layers remained consistent throughout the sample according to all analyses.

In addition to the oxide layers, other locations on the sample were attacked by Te, as shown in Figure 4(d1,e1). Compared with SS304, the density of Te-induced pitting was lower in the Fe sample, but each pit was deeper. The Te-induced pits extended to depths exceeding 50 µm beneath the sample surface, whereas in SS304, the pits typically reached a depth of about 10 µm. Within these pits, tellurium was found within the voids, as shown in Figure 4(d2,e2). In regions with concentrated Te, there was no significant enrichment of Na, O, or Fe. These observations illustrated that Te was the primary element responsible for breaching the oxide layer and penetrating the Fe substrate.

### 3.4. Nickel

**Figure 5:**
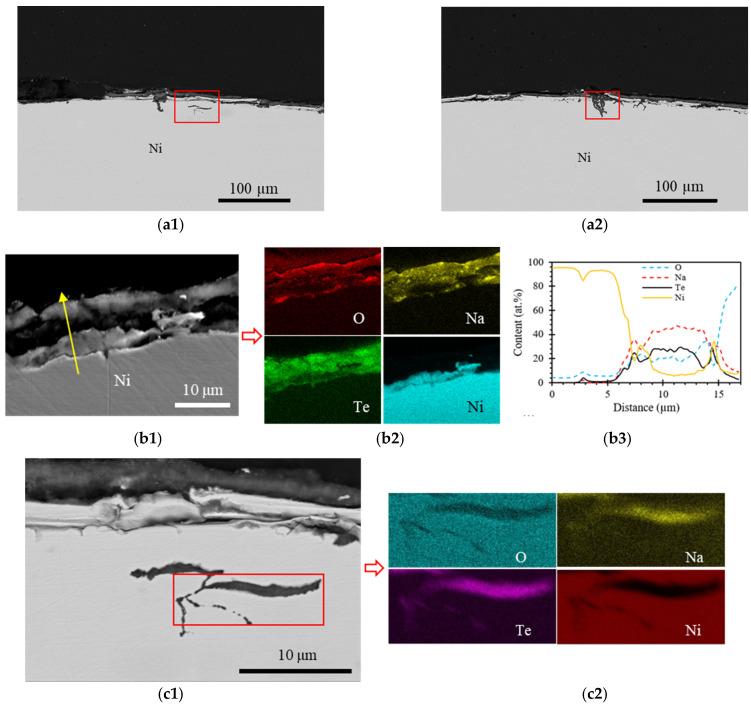

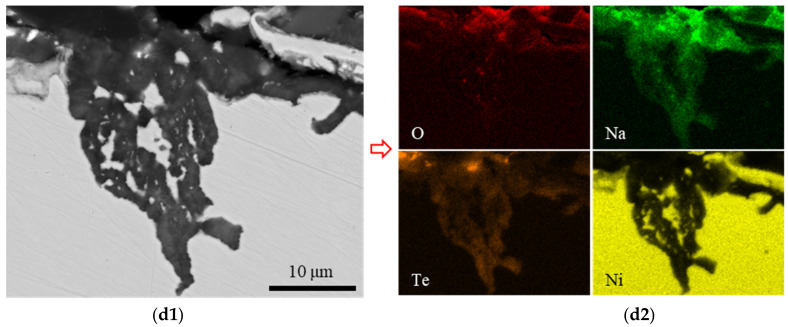
Overview cross-section SEM BSE images of post-test Ni sample (**a1**,**a2**), each with a red rectangle indicating the magnified image shown in (**c1**,**d1**). EDS mapping of elements O, Na, Te, and Ni (**c2**,**d2**) correspond to the area in (**c1**,**d1**). The oxide layer (**b1**) with EDS mapping (**b2**) and a yellow arrow representing the EDS line scan with the spectrum data in (**b3**).

The cross-section of the post-test Ni sample, with EDS mapping and a line spectrum, is shown in Figure 5. The oxide layer thickness measured around 10 µm, which is consistent with the oxide layer of the Fe sample while being thinner than the oxide layer of the SS304 sample. This oxide layer was composed of O, Na, Te, and Ni. The content of Te in the oxide layer was about 30 at. %, distinguishing it from SS304 and Fe samples, where the oxide layers contained only trace amounts of Te. When transitioning from the Ni side to the liquid sodium side, the oxide layer initially experienced an increase in the contents of O, Na, and Te, followed by stabilization, while the Ni content initially decreased and then stabilized. These observations suggest the inward diffusion of O, Na, and Te, and the outward diffusion of Ni. These characteristics of oxide layers remained consistent throughout the sample, according to all analyses.

In addition to the oxide layers, other locations on the sample were attacked by Te. The Te-induced pitting was densified across the sample surface with varying depths. Some pits extended to a depth of 30 µm into the Ni substrate, as shown in Figure 5(c1,d1). Meanwhile, smaller pits, measuring less than 10 µm in depth, were more densely distributed than their larger counterparts, as shown in Figure 5(a1,a2). As observed in the other samples, tellurium was detected within the voids of these pit defects, as evidenced by the EDS mapping.

The oxide layers and Te-induced pitting characteristics of samples as aforementioned are summarized in Table 2.

## 4. Discussion

### 4.1. Oxide Layers

Bhat and Borgstedt [11] derived the oxygen potentials of metals as functions of temperature for specific metals calculated from the free energy of formation data for binary metal oxides (e.g., Na_2_O, Cr_2_O_3_, FeO, and NiO) and oxygen solubility data for the metals (e.g., Na, Cr, Fe, and Ni). The oxygen potentials of unsaturated metal solutions in Na, Cr, Fe, and Ni are represented as follows:

(1) $$\mu_{O_{2}}^{\mathrm{Na}}\left(J/\mathrm{mol}\right)=-749470+43.24T+38.286T\ln C_{O}^{\mathrm{Na}}$$

(2) $$\mu_{O_{2}}^{\mathrm{Cr}}\left(J/\mathrm{mol}\right)=-484727-60.92T+38.286T\ln C_{O}^{\mathrm{Cr}}$$

(3) $$\mu_{O_{2}}^{\mathrm{Fe}}\left(J/\mathrm{mol}\right)=-414251-59.93T+38.286T\ln C_{O}^{\mathrm{Fe}}$$

(4) $$\mu_{O_{2}}^{\mathrm{Ni}}\left(J/\mathrm{mol}\right)=-489662+105.74T+38.286T\ln C_{O}^{\mathrm{Ni}}$$

At the test temperature of 773 K, the oxygen potentials of these metals are listed in the Table 3. At either of the two oxygen concentrations, the oxygen potential of unsaturated oxygen solution in liquid sodium is consistently more negative than the values in solid metals such as Cr, Fe, and Ni. Consequently, oxygen has a preference for entering the liquid sodium. This preference is substantiated by the EDS mapping of SS304, Fe, and Ni, where O and Na are enriched, while Cr, Fe, and Ni are either diluted or depleted.

Bhat and Borgstedt [11] also derived the oxygen potentials for the formation of ternary oxides (NaCrO_2_, Na_4_FeO_3_, and Na_2_NiO_2_) in the metals Cr, Fe, and Ni, expressed as follows:

(5) $$\mu_{O_{2}}^{\mathrm{Cr}}\left(J/\mathrm{mol}\right)=-840935+178.02T$$

(6) $$\mu_{O_{2}}^{\mathrm{Fe}}\left(J/\mathrm{mol}\right)=-812772+236.68T$$

(7) $$\mu_{O_{2}}^{\mathrm{Ni}}\left(J/\mathrm{mol}\right)=-640445+221.75T$$

As the alloying elements in SS304 do not exhibit unit activity, the oxygen potential of each alloying element in SS304 is different to the oxygen potential of pure metal. However, the extent of this change is negligible, and, as such, this factor is not addressed in this study.

**Figure 6:**
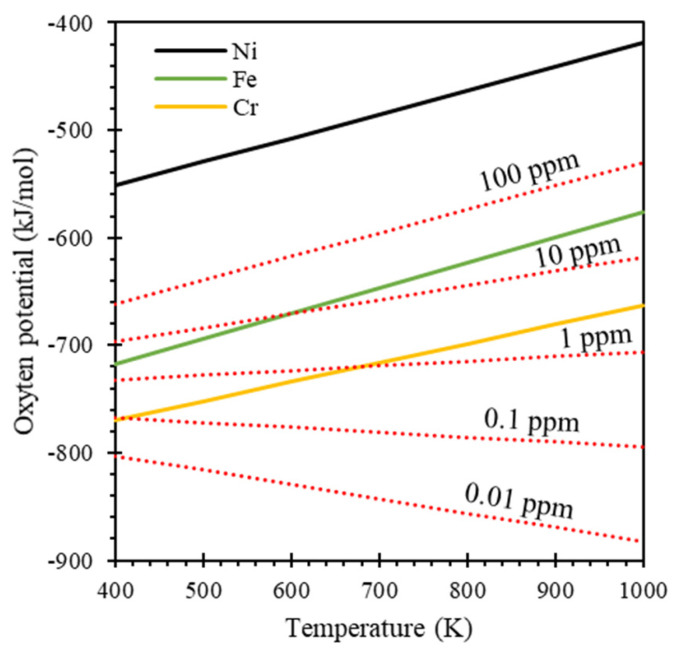
Temperature dependence of oxygen potentials for the formation of ternary oxides of metals (solid lines) compared to oxygen potentials in liquid sodium (dashed lines).

Figure 6 shows that the temperature dependence of oxygen potentials for the formation of the oxides expressed in the Equations (1) and (5)–(7). It indicates that the ternary compounds NaCrO_2_ and Na_4_FeO_3_ can only form at 773 K if the oxygen level in liquid sodium is higher than approximately 3 ppm and 30 ppm, respectively. However, the ternary compound Na_2_NiO_2_ cannot be formed, as the threshold oxygen level is extraordinarily high.

The results in this study are highly consistent with the aforementioned theory. In the SS304 sample (with an oxygen level of 10 ppm under the test condition), as shown in Figure 1, oxide layers were formed through the interdiffusion of O and Na from the liquid sodium side and Cr, Fe, and Ni from the SS304 side. The diffusion behavior of Cr was far different from that of Fe and Ni. Chromium was enriched in layer-3, which was nearly depleted of Fe and Ni, while Cr was depleted in layer-4, which was highly enriched with Fe and Ni. The composition of layer-3 was roughly 25(Na,Fe)-25Cr-50O at.%, which is close to the composition of ternary compound NaCrO_2_. The Fe atoms substituted Na atoms in the composition, likely due to the outward diffusion Fe from the SS304 side and the measurement uncertainty of EDS measurements in a small detection area. This finding aligns with a previous study, where NaCrO_2_ was found even at extremely low oxygen levels (20–6200 ppm, 873 K) [10]. The composition of layer-4 was roughly 80Fe-10Ni-10O at.%, which does not indicate the presence of any metal–sodium oxide compounds (e.g., Na_2_NiO_2_ and Na_4_FeO_3_). This metal oxide layer is prone to reduction to pure metal. However, the evolution of this metal oxide layer over time is beyond the scope of this study and is not discussed.

In contrast, the Cr sample (0.01 ppm oxygen level under the test condition), did not exhibit the formation of a NaCrO_2_ oxide layer. This absence is attributed to the fact that the oxygen level during testing was much lower than the threshold required for the formation of the ternary oxide NaCrO_2_. Similarly, the Fe and Ni samples, which also had oxygen levels of 0.01 ppm, did not exhibit Na_2_NiO_2_ and Na_4_FeO_3_ oxide layers, either, due to the extremely low oxygen levels during testing. However, it was noted that about 10 μm thick oxide layers did form in the Fe and Ni samples, despite the low oxygen levels, with the compositions of 40Na-40Fe-20O at.% and 40Na-10Ni-30Te-20O at.%, respectively. These oxide layers were a result of the interdiffusion between the elements present in the materials.

### 4.2. Tellurium Penetration

The Te-induced pitting characteristics reveal a preference for attacking Ni and Fe substrates over Cr substrate. Similar phenomena have been found in previous studies, as aforementioned, in which Te reacted with Ni and Fe [7,8], while its effects on Cr were limited by the formation of Cr_3_Te_4_ scale [9]. Metals would react with Te, forming tellurides, and the thermodynamic stability of the tellurides, Cr_3_Te_4_, TeFe, and NiTe, can be ranked from more to less stable as follows: Cr_3_Te_4_, TeFe, and NiTe. The enthalpy of formation at 298 K for these tellurides is as follows: Cr_3_Te_4_ is −34.5 kJ/g-atom [15], TeFe is −10 kJ/g-atom [16], and NiTe is −8 kJ/g-atom [16].

Given that Cr_3_Te_4_ is relatively more stable, forming this telluride scale may inhibit further chemical reactions between Cr and Te. Conversely, the reaction products, TeFe and NiTe, might be less stable, and thus dissolve into the liquid sodium. This dissolution can induce additional chemical reactions between Te and Ni, as well as Te and Fe, resulting in a deeper pitting. In the case of the SS304 sample, it also exhibits relatively shallow pitting, similar to the Cr sample, but with a much higher pitting density. This can be attributed to the presence of Cr in SS304, which restricts Te diffusion deep into the steel substrate; additionally, the susceptible NaCrO_2_ layer (i.e., layer-3 in Figure 1) served as a protective barrier. The densely distributed pits across the sample surface were due to the breakdown of the Fe-Ni-oxide layer (i.e., layer-4 in Figure 1). EDS mapping of SS304 in Figure 2(b2) showed that the Fe and Ni compositions adjacent to the pits on the liquid sodium side were higher than the steel side, opposite to the Cr composition. This observation suggests that the oxide layer disintegrated, with Fe and Ni dissolving into liquid sodium. Meanwhile, the presence of Cr impeded further reaction between Te and the substrate.

## 5. Conclusions

The Te-induced corrosion behavior of structural material SS304 and its main alloying elements, Fe, Cr, and Ni, in a high-temperature liquid sodium environment, was studied. The research yielded the following significant findings:

- The oxygen level emerges as a critical factor in the formation of stable oxide compound layers, which play a crucial role in mitigating Te-induced pitting to some extent. When the oxygen level was maintained at 10 ppm, multiple oxide layers formed on the surface of SS304. These layers consisted of a compact NaCrO_2_ thin interlayer and porous outer layers comprising Na-Fe-Ni-O. Notably, this oxygen level exceeded the threshold for NaCrO_2_ formation but remained below the levels required for Na_4_FeO_3_ and Na_2_NiO_2_. The compact NaCrO_2_ thin interlayer was effective in hindering Te penetration, unlike porous oxide layers. At an oxygen level of 0.01 ppm, stable ternary oxide compounds failed to form.
- Chromium had greater resistance to Te-induced pitting when compared to other alloying elements, Fe and Ni. The depth of Te-induced pitting was greater in Fe and Ni compared to Cr. This difference resulted from the lower thermodynamic stability of Fe- and Ni-tellurides when compared to Cr-tellurides. Consequently, Fe- and Ni-tellurides were more prone to dissolution in the liquid sodium, while Cr-tellurides acted as a barrier against further tellurium-induced pitting.

In summary, this investigation offers valuable insights into the corrosion behavior of structural materials in high-temperature liquid sodium environments, particularly in the presence of Te. Understanding these behaviors is crucial for enhancing the safety and performance of SFRs, and further systematic research in this area is desired.

## Figures and Tables

**Table 1:** Summary of test materials and conditions.

Test Material	Test Condition		
	Test Time (d)	Glovebox Oxygen Level (ppm)	Liquid Condition
SS304	30	10	1 mol.% Te in liquid sodium at 773 K
Cr	30	0.01	
Fe	30	0.01	
Ni	30	0.01	

**Table 2:** Summary of oxide layer and Te-induced pitting characteristics.

Test Material	Glovebox Oxygen Level (ppm)	Oxide Layer Characteristics	Te-Induced Pitting Characteristics
SS304	10	10–20 µm thick, no Te composition, multiple layers with various compositions	Densified pits of 10 µm depth
Cr	0.01	No visible oxide layer	A few pits of 5–10 µm depth
Fe	0.01	10 µm thick with a little Te composition, single layer	A few pits of 50 µm depth
Ni	0.01	10 µm thick with about 30 at.% Te, single layer	Densified pits of 10–30 µm depth

**Table 3:** Oxygen potentials for the formation of binary metal oxides at 773 K.

Metal	$\mathrm{Oxygen}\;\mathrm{Concentration}\;C_{O}\;\left(ppm\right)$	$\mathrm{Oxygen}\;\mathrm{Potential}\;\mu_{O_{2}}\;\left(J/mol\right)$	$\mathrm{Oxygen}\;\mathrm{Concentration}\;C_{O}\;\left(ppm\right)$	$\mathrm{Oxygen}\;\mathrm{Potential}\;\mu_{O_{2}}\;\left(J/mol\right)$
Na → Na_2_O	10	−647,900	0.01	−852,336
Cr → Cr_2_O_3_		−463,673		−668,109
Fe → FeO		−392,432		−596,867
Ni → NiO		−339,780		−544,215

## Data Availability

The data supporting the findings of the study is available upon request. Interested parties may contact the corresponding author to obtain the data.

## References

[1] Middleton B.D., Parma E.J., Olivier T.J., Phillips J., Lachance J.L. (2011). The Development of a Realistic Source Term for Sodium-Cooled Fast Reactors: Assessment of Current Status and Future Needs.

[2] (1993). Fission and Corrosion Product Behaviour in Liquid Metal Fast Breeder Reactors (LMFBRs).

[3] Grabaskas D., Brunett A., Bucknor M., Sienicki J., Sofu T. (2015). Regulatory Technology Development Plan Sodium Fast Reactor Mechanistic Source Term Development.

[4] Feurestein H., Hooper A., Johnson F. (1979). Mechanisms of Release of Radioactive Products into Liquid-Metal Coolants, their Transport within the Circuits and Removal from LMFBRs. At. Energy Rev..

[5] Chellew N.R., Bennett G.A. (1961). The Melt Refining of Irradiated Uranium: Application to EBR-II Fast Reactor Fuel. XII. The Behavior of Ruthenium, Molybdenum, Palladium, Rhodium, Technetium, Antimony, Cadmium, and Tellurium. Nucl. Sci. Eng..

[6] Zhang Z.-D., Ren C.-L., Tan M.-L., Yang Y.-Q., Yin Y.-R., Wang C.-Y., Han H., Huai P. (2020). Migration behavior of tellurium in bcc iron against typical alloying elements: A first-principles study. Comput. Mater. Sci..

[7] Rosenthal M.W., Kasten P.R., Briggs R.B. (2017). Molten-Salt Reactors—History, Status, and Potential. Nucl. Appl. Technol..

[8] Jia Y., Cheng H., Qiu J., Han F., Zou Y., Li Z., Zhou X., Xu H. (2013). Effect of temperature on diffusion behavior of Te into nickel. J. Nucl. Mater..

[9] Wu B.H., Jiang L., Ye X.X., Li C.W., Liang J.P., Liu F., Li Z.J. (2020). On the origin of tellurium corrosion resistance of hot-rolled GH3535 alloy. Corros. Sci..

[10] Barker M.G., Wood D.J. (1974). The corrosion of chromium, iron, and stainless steel in liquid sodium. J. Less Common Met..

[11] Bhatt N.P., Borgstedt H.U. (1988). Corrosion behaviour of structural materials in sodium influenced by formation of ternary oxides. Mater. Corros..

[12] Walker R.A., Pratt J.N. (1970). The solubilities of bismuth and tellurium in liquid sodium. J. Nucl. Mater..

[13] Borgstedt H.U., Guminski C. (2000). Solubilities and Solution Chemistry in Liquid Alkali Metals. Monatshefte Für Chem./Chem. Mon..

[14] Noden J.D. (1973). A general equation for the solubility of oxygen in liquid sodium. J. Br. Nucl. Energy Soc..

[15] Grønvold F., Westrum E.F. (1964). Thermodynamic aspects of the magnetic transitions in the chromium tellurides Heat Capacities of Cr_5_Te_6_, Cr_3_Te_4_ and Cr_2_Te_3_ from 5 to 350 K. Z. Für Anorg. Und Allg. Chem..

[16] Arvhult C.M., Guéneau C., Gossé S., Selleby M. (2018). Thermodynamic assessment of the Fe-Te system. Part II: Thermodynamic modeling. J. Alloys Compd..

